# Nonreciprocal reconfigurable microwave optomechanical circuit

**DOI:** 10.1038/s41467-017-00447-1

**Published:** 2017-09-19

**Authors:** N. R. Bernier, L. D. Tóth, A. Koottandavida, M. A. Ioannou, D. Malz, A. Nunnenkamp, A. K. Feofanov, T. J. Kippenberg

**Affiliations:** 1 Institute of Physics, École Polytechnique Fédérale de Lausanne, Lausanne, 1015 Switzerland; 2 Cavendish Laboratory, University of Cambridge, Cambridge, CB3 0HE UK

## Abstract

Nonreciprocal microwave devices are ubiquitous in radar and radio communication and indispensable in the readout chains of superconducting quantum circuits. Since they commonly rely on ferrite materials requiring large magnetic fields that make them bulky and lossy, there has been significant interest in magnetic-field-free on-chip alternatives, such as those recently implemented using the Josephson nonlinearity. Here, we realize reconfigurable nonreciprocal transmission between two microwave modes using purely optomechanical interactions in a superconducting electromechanical circuit. The scheme relies on the interference in two mechanical modes that mediate coupling between the microwave cavities and requires no magnetic field. We analyse the isolation, transmission and the noise properties of this nonreciprocal circuit. Finally, we show how quantum-limited circulators can be realized with the same principle. All-optomechanically mediated nonreciprocity demonstrated here can also be extended to directional amplifiers, and it forms the basis towards realizing topological states of light and sound.

## Abstract (web summary)

Nonreciprocal optical devices traditionally rely on magnetic fields and magnetic-free approaches are rather recent. Here, Bernier et al. propose and demonstrate a purely optomechanical circulator with reconfigurable transmission without the need for direct coupling between input and output modes.

## Introduction

Nonreciprocal devices, such as isolators, circulators and directional amplifiers, exhibit altered transmission characteristics if the input and output channels are interchanged. They are essential to several applications in signal processing and communication, as they protect devices from interfering signals^1^. At the heart of any such device lies an element breaking Lorentz reciprocal symmetry for electromagnetic sources^2, 3^. Such elements have included ferrite materials^4–6^, magneto-optical materials^7–10^, optical nonlinearities^11–13^, temporal modulation^14–19^, chiral atomic states^20^ and physical rotation^21^. Typically, a commercial nonreciprocal microwave apparatus exploits ferrite materials and magnetic fields, which leads to a propagation-direction-dependent phase shift for different field polarizations. A significant drawback of such devices is that they are ill-suited for sensitive superconducting circuits, since their strong magnetic fields are disruptive and require heavy shielding. In recent years, the major advances in quantum superconducting circuits^22^, that require isolation from noise emanating from readout electronics, have led to a significant interest in nonreciprocal devices operating at the microwave frequencies that dispense with magnetic fields and can be integrated on-chip.

As an alternative to ferrite-based nonreciprocal technologies, several approaches have been pursued towards nonreciprocal microwave chip-scale devices. Firstly, the modulation in time of the parametric couplings between modes of a network can simulate rotation about an axis, creating an artificial magnetic field^14, 18, 23, 24^ rendering the system nonreciprocal with respect to the ports. Secondly, phase matching of a parametric interaction can lead to nonreciprocity, since the signal only interacts with the pump when copropagating with it and not in the opposite direction. This causes travelling-wave amplification to be directional^24–27^. Phase-matching-induced nonreciprocity can also occur in optomechanical systems^28, 29^, where parity considerations for the interacting spatial modes apply^30–32^. Finally, interference in parametrically coupled multi-mode systems can be used. In these systems, nonreciprocity arises due to interference between multiple coupling pathways along with dissipation in ancillary modes^33^. Here, dissipation is a key resource to break reciprocity, as it forms a flow of energy always leaving the system, even as input and output are interchanged. It has therefore been viewed as reservoir engineering^34^. Following this approach, nonreciprocity has recently been demonstrated in Josephson-junctions-based microwave circuits^35, 36^ and in a photonic-crystal-based optomechanical circuit^37^. These realizations and theoretical proposals to achieve nonreciprocity in multi-mode systems rely on a direct, coherent coupling between the electromagnetic input and output modes.

Here, in contrast, we describe a scheme to attain reconfigurable nonreciprocal transmission without a need for any direct coherent coupling between input and output modes, using purely optomechanical interactions^28, 29^. This scheme neither requires cavity–cavity interactions nor phonon–phonon coupling, which are necessary for the recently demonstrated optomechanical nonreciprocity in the optical domain^37^. Two paths of transmission between the microwave modes are established, through two distinct mechanical modes. Interference between those paths with differing phases forms the basis of the nonreciprocal process^38, 39^. In fact, due to the finite quality factor of the intermediary mechanical modes, both conversion paths between the electromagnetic modes are partly dissipative in nature. Nonreciprocity is in this case only possible by breaking the symmetry between the two dissipative coupling pathways. We describe the mechanism in detail below, shedding some light on the essential ingredients for nonreciprocity using this approach.

## Results

### Theoretical model

**Fig. 1:**
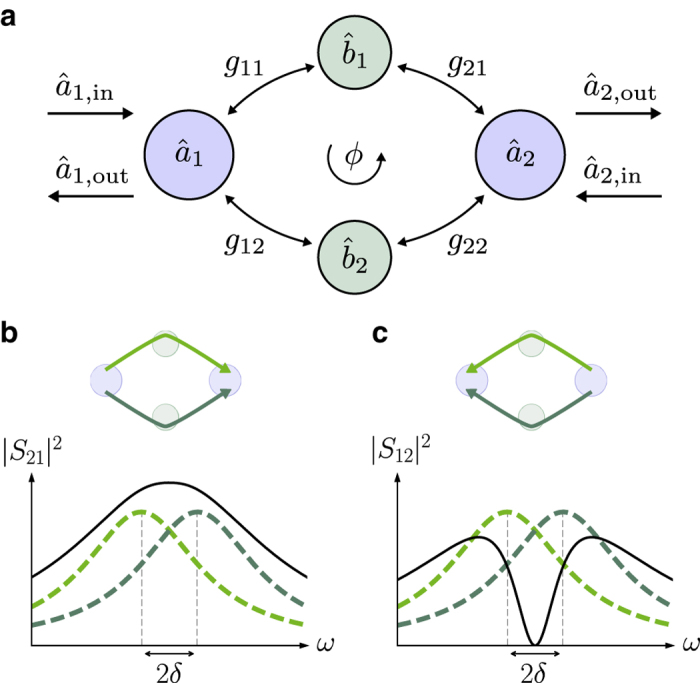
Optomechanical nonreciprocal transmission via interference of two asymmetric dissipative coupling pathways. **a** Two microwave modes ${{\hat a}_1}$ and ${{\hat a}_2}$ are coupled via two mechanical modes ${\hat b_1}$ and ${\hat b_2}$ through optomechanical frequency conversion (as given by the coupling constants *g*_11_, *g*_21_, *g*_12_, *g*_22_). Nonreciprocity is based on the interference between the two optomechanical (conversion) pathways *g*_11_, *g*_21_ and *g*_12_, *g*_22_, in the presence of a suitably chosen phase difference *ϕ* between the coupling constants as well as the deliberate introduction of an asymmetry in the pathways. **b**, **c** The symmetry between the pathways can be broken by off-setting the optomechanical transmission windows through each mechanical mode (*dashed lines* in *dark* and *light green*) by a frequency difference 2*δ*, resulting in different |*S*_21_| and |*S*_12_| (*solid lines*). Each single pathway, in the absence of the other mode, is described by Eq. (2). In the forward direction **b**, the two paths interfere constructively, allowing transmission and a finite scattering matrix element *S*_21_ on resonance with the first microwave cavity. In contrast, in the backward direction **c**, the paths interfere destructively, such that *S*_12_ ≈ 0, thereby isolating port 1 from port 2 on resonance with the second microwave cavity. The isolation bandwidth is determined by the intrinsic dissipation rate of the mechanical modes

**Fig. 2:**
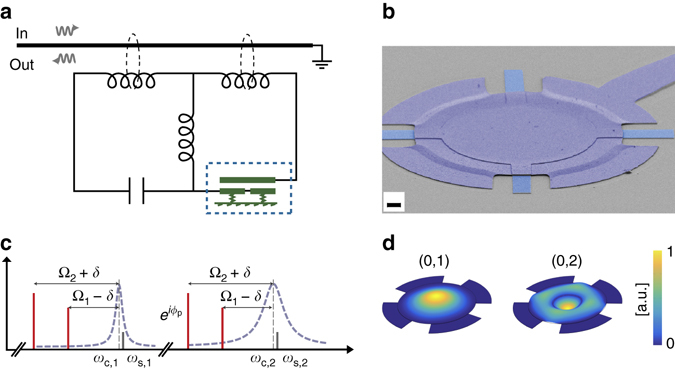
Implementation of a superconducting microwave circuit optomechanical device for nonreciprocity. **a** A superconducting circuit featuring two electromagnetic modes in the microwave domain is capacitively coupled to a mechanical element (a vacuum-gap capacitor, *dashed rectangle*) and inductively coupled to a microstrip feedline. The end of the feedline is grounded and the circuit is measured in reflection. **b** Scanning electron micrograph of the drum-head-type vacuum gap capacitor (*dashed rectangle* in **a**) with a gap distance below 50 nm, made from aluminium on a sapphire substrate. The scale bar indicates 2 μm. **c** Frequency domain schematic of the microwave pump setup to achieve nonreciprocal mode conversion. Microwave pumps (*red bars*) are placed at the lower motional sidebands—corresponding to the two mechanical modes—of both microwave resonances (*dashed purple lines*). The pumps are detuned from the exact sideband condition by ±*δ* = 2*π* ⋅ 18 kHz, creating two optomechanically induced transparency windows detuned by 2*δ* from the microwave resonance frequencies (denoted by *ω*_c,1_ and *ω*_c,2_, *vertical dashed lines*). The phase *ϕ*_p_ of one the pumps is tuned. The propagation of an incoming signal (with frequency *ω*_s,1_ or *ω*_s,2_, *solid grey bars*) in the forward and backward direction depends on this phase and nonreciprocal microwave transmission can be achieved. **d** Finite-element simulation of the displacement of the fundamental (0, 1) and second order radially symmetric (0, 2) mechanical modes (with measured resonant frequencies Ω_1_/2*π* = 6.5 MHz and Ω_2_/2*π* = 10.9 MHz, respectively) which are exploited as intermediary dissipative modes to achieve nonreciprocal microwave conversion

We first theoretically model our system to reveal how nonreciprocity arises. We consider two microwave modes (described by their annihilation operators ${\hat a}_1$, ${\hat a}_2$) having resonance frequencies *ω*_c,1_, *ω*_c,2_ and dissipation rates *κ*_1_, *κ*_2_, which are coupled to two mechanical modes (described by the annihilation operators ${\hat b_1}$, ${\hat b_2}$) having resonance frequencies Ω_1_, Ω_2_ and dissipation rates Γ_m,1_, Γ_m,2_ (Fig. 1a). The radiation-pressure-type optomechanical interaction has the form^28, 29^ ${g_{0,ij}}{\hat a}_i^\dag {\hat a}_i({\hat b}_j + {\hat b}_j^\dag )$ (in units where *ħ* = 1), where *g*_0,*ij*_ designates the vacuum optomechanical coupling strength of the *i* ^th^ microwave mode to the *j* ^th^ mechanical mode. Four microwave tones are applied, close to each of the two lower sidebands of the two microwave modes, with detunings of Δ_11_ = Δ_21_ = −Ω_1_ − *δ* and Δ_12_ = Δ_22_ = −Ω_2_ + *δ* (Fig. 2c). We linearize the Hamiltonian, neglect counter-rotating terms, and write it in a rotating frame with respect to the mode frequencies (Supplementary Note 1)

1 $$\begin{array}{ccccc} H & = - \delta \,\hat b_1^\dag {{\hat b}_1} + \delta \,\hat b_2^\dag {{\hat b}_2} + {g_{11}}({{\hat a}_1}\hat b_1^\dag + {\hat a}_1^\dag {{\hat b}_1}) + {g_{21}}({{\hat a}_2}\hat b_1^\dag + {\hat a}_2^\dag {{\hat b}_1})\\ & + {g_{12}}({{\hat a}_1}\hat b_2^\dag + {\hat a}_1^\dag {{\hat b}_2}) + {g_{22}}({e^{i\phi }}{{\hat a}_2}\hat b_2^\dag + {e^{ - i\phi }}{\hat a}_2^\dag {{\hat b}_2}) \end{array}$$

where ${\hat a}_i$ and ${\hat b_j}$ are redefined to be the quantum fluctuations around the linearized mean fields. Here ${g_{ij}} = {g_{0,ij}}\sqrt {{n_{ij}}}$ are the field-enhanced optomechanical coupling strengths, where *n*_*ij*_ is the contribution to the mean intracavity photon number due to the drive with detuning Δ_*ij*_. Although in principle each coupling is complex, without loss of generality we can take all to be real except the one between ${\hat a}_2$ and ${\hat b_2}$ with a complex phase *ϕ*.

We start by considering frequency conversion through a single mechanical mode. Neglecting the noise terms, the field exiting the cavity ${\hat a}_2$ is given by ${\hat a}_{2,{\rm{out}}} = {S_{21}}{\hat a}_{1,{\rm{in}}} + {S_{22}}{{\hat a}_{2,{\rm{in}}}}$, which defines the scattering matrix *S*_*ij*_. For a single mechanical pathway, setting *g*_12_ = *g*_22_ = 0 and *δ* = 0, the scattering matrix between input and output mode becomes

2 $${S_{21}}(\omega ) = \sqrt {\frac{{{\kappa _{{\rm{ex}},1}}{\kappa _{{\rm{ex}},2}}}}{{{\kappa _1}{\kappa _2}}}} \frac{{\sqrt {{{\cal C}_{11}}{{\cal C}_{21}}} {\Gamma _{{\rm{m,1}}}}}}{{\frac{{{\Gamma _{{\rm{eff,1}}}}}}{2} - i\omega }},$$

where *κ*_ex,1_, *κ*_ex,2_ denote the external coupling rates of the microwave modes to the feedline, and the (multiphoton) cooperativity for each mode pair is defined as ${{\cal C}_{ij}} = 4g_{ij}^2{\rm{/}}({\kappa _i}{\Gamma _{{\rm{m}},j}})$. Conversion occurs within the modified mechanical response over an increased bandwidth ${\Gamma _{{\rm{eff,1}}}} = {\Gamma _{{\rm{m,1}}}}\left( {1 + {{\cal C}_{11}} + {{\cal C}_{21}}} \right)$. This scenario, where a mechanical oscillator mediates frequency conversion between electromagnetic modes, has recently been demonstrated^40^ with a microwave optomechanical circuit^41^, and moreover used to create a bidirectional link between a microwave and an optical mode^42^. Optimal conversion, limited by internal losses in the microwave cavities, reaches at resonance $\left| {{S_{21}}} \right|_{{\rm{max}}}^2 = \frac{{{\kappa _{{\rm{ex}},1}}{\kappa _{{\rm{ex}},2}}}}{{{\kappa _1}{\kappa _2}}}$ in the limit of large cooperativities ${{\cal C}_{11}} = {{\cal C}_{21}} \gg 1$.

We next describe the nonreciprocal transmission of the full system with both mechanical modes. We consider the ratio of transmission amplitudes given by

3 $$\frac{{{S_{12}}(\omega )}}{{{S_{21}}(\omega )}} = \frac{{{g_{11}}{\chi _1}(\omega ){g_{21}} + {g_{12}}{\chi _2}(\omega ){g_{22}}{e^{ + i\phi }}}}{{{g_{11}}{\chi _1}(\omega ){g_{21}} + {g_{12}}{\chi _2}(\omega ){g_{22}}{e^{ - i\phi }}}}$$

with the mechanical susceptibilities defined as $\chi _1^{ - 1}(\omega ) = {\Gamma _{{\rm{m,1}}}}{\rm{/}}2 - i\left( {\delta + \omega } \right)$ and $\chi _2^{ - 1}(\omega ) = {\Gamma _{{\rm{m,2}}}}{\rm{/}}2 + i\left( {\delta - \omega } \right)$. Conversion is nonreciprocal if the above expression has a magnitude that differs from 1. If *S*_21_ and *S*_12_ differ only by a phase, it can be eliminated by a redefinition of either ${{\hat a}_1}$ or ${{\hat a}_2}$ ^24, 33^. Upon a change in conversion direction, the phase *ϕ* of the coherent coupling (between the microwave and mechanical mode) is conjugated, while the complex phase associated with the response of the dissipative mechanical modes remains unchanged. Physically, scattering from 1 → 2 is related to scattering from 2 → 1 via time-reversal, which conjugates phases due to coherent evolution of the system. Dissipation is untouched by such an operation and thus remains invariant. Indeed, the mechanical dissipation is an essential ingredient for the nonreciprocity to arise in this system, but not sufficient on its own. In fact, if we align the frequency conversion windows corresponding to the two mechanical modes by setting *δ* = 0, the system becomes reciprocal on resonance (*ω* = 0), since there is no longer any phase difference between numerator and denominator. This situation corresponds to two symmetric pathways resulting from purely dissipative couplings; they can interfere only in a reciprocal way.

### Conditions for isolation

We study the conditions for isolation when backward transmission *S*_12_ vanishes while forward transmission *S*_21_ is non-zero. A finite offset 2*δ* between the mechanical conversion windows causes an intrinsic phase shift for a signal on resonance (*ω* = 0) travelling one path compared to the other, as it falls either on the red or the blue side of each mechanical resonance. The coupling phase *ϕ* is then adjusted to cancel propagation in the backward direction *S*_12_ (Fig. 1c), by cancelling the two terms in the numerator of Eq. (3). In general, there is always a frequency *ω* for which |*g*_11_ *χ*_1_(*ω*)*g*_21_| = |*g*_12_ *χ*_2_(*ω*)*g*_22_|, such that the phase *ϕ* can be tuned to cancel transmission in one direction. Specifically, for two mechanical modes with identical decay rates (Γ_m,1_ = Γ_m,2_ = Γ_m_) and symmetric couplings (*g*_11_ *g*_21_ = *g*_12_ *g*_22_), we find that transmission from ports 2 to 1 vanishes on resonance if

4 $$\frac{{{\Gamma _{\rm{m}}}}}{{2\delta }} = {\rm{tan}}\frac{\phi }{2}.$$

The corresponding terms of the denominator will have a different relative phase, and the signal will add constructively instead, in the forward direction (Fig. 1b). The device thus acts as an isolator from ${{\hat a}_1}$ to ${{\hat a}_2}$, realized without relying on the Josephson nonlinearity^35, 36^. We now describe the conditions to minimize insertion loss of the isolator in the forward direction. Still considering the symmetric case, the cooperativity is set to be the same for all modes (${{\cal C}_{ij}} = {\cal C}$). For a given separation *δ*, transmission on resonance (*ω* = 0) in the isolating direction has the maximum

5 $$\left| {{S_{21}}} \right|_{{\rm{max}}}^{\rm{2}} = \frac{{{\kappa _{{\rm{ex}},1}}{\kappa _{{\rm{ex}},2}}}}{{{\kappa _1}{\kappa _2}}}\left( {1 - \frac{1}{{2{\cal C}}}} \right)$$

for a cooperativity ${\cal C} = 1{\rm{/}}2 + 2{\delta ^2}{\rm{/}}\Gamma _{\rm{m}}^2$. As in the case for a single mechanical pathway in Eq. (2), for large cooperativity, the isolator can reach an insertion loss only limited by the internal losses of the microwave cavities.

The unusual and essential role of dissipation in this nonreciprocal scheme is also apparent in the analysis of the bandwidth of the isolation. Although the frequency conversion through a single mechanical mode has a bandwidth Γ_eff,*j*_ (Eq. (2)), caused by the optomechanical damping of the pumps on the lower sidebands, the nonreciprocal bandwidth is set by the intrinsic mechanical damping rates. Examination of Eq. (3) reveals that nonreciprocity originates from the interference of two mechanical susceptibilities of widths Γ_m,*j*_. One can conclude that the intrinsic mechanical dissipation, which takes energy out of the system regardless of the transmission direction, is an essential ingredient for the nonreciprocal behaviour reported here, as discussed previously^33, 34^. In contrast, optomechanical damping works symmetrically between input and output modes. By increasing the coupling rates, using higher pump powers, the overall conversion bandwidth increases, while the nonreciprocal bandwidth stays unchanged.

### Experimental realization

We experimentally realize this nonreciprocal scheme using a superconducting circuit optomechanical system in which mechanical motion is capacitively coupled to a multimode microwave circuit^41^. The circuit, schematically shown in Fig. 2a, supports two electromagnetic modes with resonance frequencies (*ω*_c,1_, *ω*_c,2_) = 2*π* ⋅ (4.1, 5.2) GHz and energy decay rates (*κ*_1_, *κ*_2_) = 2*π* ⋅ (0.2, 3.4) MHz, both of them coupled to the same vacuum-gap capacitor. We utilize the fundamental and second order radially symmetric (0, 2) modes of the capacitor’s mechanically compliant top plate^43^ (Fig. 2b, d) with resonance frequencies (Ω_1_, Ω_2_) = 2*π* ⋅ (6.5, 10.9) MHz, intrinsic energy decay rates (Γ_m,1_, Γ_m,2_) = 2*π* ⋅ (30, 10) Hz and optomechanical vacuum coupling strengths (*g*_0,11_, *g*_0,12_) = 2*π* ⋅ (91, 12) Hz, respectively (with *g*_0,11_ ≈ *g*_0,21_ and *g*_0,12_ ≈ *g*_0,22_, i.e. the two microwave cavities are symmetrically coupled to the mechanical modes). The device is placed at the mixing chamber of a dilution refrigerator at 200 mK and all four incoming pump tones are heavily filtered and attenuated to eliminate Johnson and phase noise (details are published elsewhere^44^). We establish a parametric coupling between the two electromagnetic and the two mechanical modes by introducing four microwave pumps with frequencies slightly detuned from the lower motional sidebands of the resonances, as shown in Fig. 2c and as discussed above. An injected probe signal *ω*_s1(s2)_ around the lower (higher) frequency microwave mode is then measured in reflection using a vector network analyser.

**Fig. 3:**
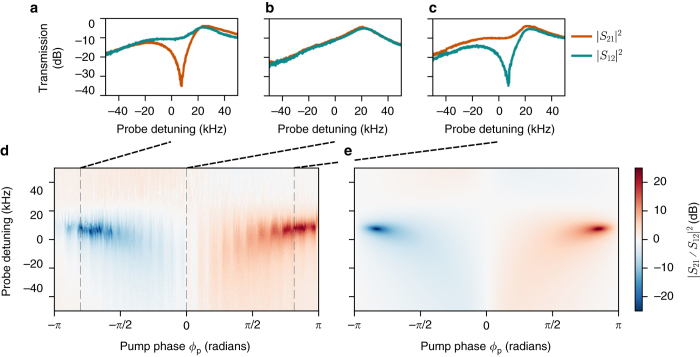
Experimental demonstration of nonreciprocity. **a**–**c** Power transmission between modes 1 and 2 as a function of probe detuning, shown in both directions for pump phases *ϕ*_p_ = −0.8*π*, 0, 0.8*π* radians (respectively **a**–**c**). Isolation of more than 20 dB in the forward **c** and backward **a** directions is demonstrated, as well as reciprocal behaviour **b**. **d** The ratio of transmission |*S*_21_/*S*_12_|^2^, representing a measure of nonreciprocity, is shown as a function of pump phase *ϕ*_p_ and probe detuning. Two regions of nonreciprocity develop, with isolation in each direction. The system is reconfigurable as the direction of isolation can be swapped by taking *ϕ*_p_ → −*ϕ*_p_. **e** Theoretical ratio of transmission from Eq. (3), calculated with independently estimated experimental parameters. The theoretical model includes effectively lowered cooperativities for the mechanical mode ${\hat b_1}$ due to cross-damping (optomechanical damping of the lower frequency mechanical mode by the pump on the sideband of the higher frequency mechanical mode) acting as an extra loss channel

Frequency conversion in both directions, |*S*_21_(*ω*)|^2^ and |*S*_12_(*ω*)|^2^, are measured and compared in Fig. 3a–c. The powers of the four pumps are chosen such that the associated individual cooperativities are given by ${{\cal C}_{11}} = 520$, ${{\cal C}_{21}} = 450$, ${{\cal C}_{12}} = 1350$ and ${{\cal C}_{22}} = 1280$. The detuning from the lower motional sidebands is set to *δ* = 2*π* ⋅ 18 kHz. By pumping both cavities on the lower sideband associated with the same mechanical mode, a signal injected on resonance with one of the modes will be frequency converted to the other mode. This process can add negligible noise, when operating with sufficiently high cooperativity, as demonstrated recently^40^. In the experiment, the four drive tones are all phase-locked and the phase of one tone *ϕ*_p_ is varied continuously from −*π* to *π*. The pump phase is linked to the coupling phase *ϕ* by a constant offset, in our case *ϕ*_p_ ≈ *ϕ* + *π*. Between the two transmission peaks corresponding to each mechanical mode, a region of nonreciprocity develops, depending on the relative phase *ϕ*_p_.

The amount of reciprocity that occurs in this process is quantified and measured by the ratio of forward to backward conversion |*S*_21_/*S*_12_|^2^. Figure 3d shows this quantity as a function of probe detuning and the relative pump phase. Isolation of more than 20 dB is demonstrated in each direction in a reconfigurable manner, i.e. the direction of isolation can be switched by taking *ϕ*_p_ → −*ϕ*_p_, as expected from Eq. (4). The ideal theoretical model, which takes into account Γ_m,1_ ≠ Γ_m,2_, predicts that the bandwidth of the region of nonreciprocity is commensurate with the arithmetic average of the bare mechanical dissipation rates, ~2*π* · 20 Hz. However, given the significantly larger coupling strength of the fundamental mechanical mode compared to the second order mode, and that *κ*_2_/Ω_1,2_ is not negligible, the pump detuned by Ω_2_ − *δ* from the microwave mode ${{\hat a}_2}$ introduces considerable cross-damping (i.e. resolved sideband cooling) for the fundamental mode. This cross-damping, measured separately to be $\Gamma _{{\rm{m,1}}}^{{\rm{(cross)}}} \approx 2\pi \, \cdot \,20$ kHz at the relevant pump powers, widens the bandwidth of nonreciprocal behaviour by over two orders of magnitude and effectively cools the mechanical oscillator. It also acts as a loss in the frequency conversion process and therefore effectively lowers the cooperativities to $({{\cal C}_{11}},{{\cal C}_{21}}) \approx (0.78,0.68)$. This lowered cooperativity accounts for the overall ~10 dB loss in the forward direction. This limitation can be overcome in a future design by increasing the sideband resolution with decreased *κ*_*i*_ or utilising the fundamental modes of two distinct mechanical elements with similar coupling strengths. To compare the experiment to the theory we use a model that takes into account the cross-damping and an increased effective mechanical dissipation of the fundamental mode. The model is compared to the experimental data in Fig. 3e, showing good qualitative agreement.

### Noise properties

From a technological standpoint, it does not suffice for an isolator to have the required transmission properties; since its purpose is to protect the input from any noise propagating in the backward direction, the isolator’s own noise emission is relevant. We, therefore, return to the theoretical description of the ideal symmetric case and derive the noise properties expected from the device, in the limit of overcoupled cavities (*κ*_ex,*i*_ ≈ *κ*_*i*_). In the forward direction and on resonance, the emitted noise amounts to ${N_{{\rm{fw}}}}(0) = 1{\rm{/2}} + ({\bar n_{{\rm{m}},1}} + {\bar n_{{\rm{m}},2}}){\rm{/}}(4{\cal C})$, where ${\bar n_{{\rm{m}},j}}$ is the thermal occupation of each mechanical mode (Supplementary Note 2). In the limit of low insertion loss and large cooperativity, the added noise becomes negligible in the forward direction. More relevant for the purpose of using an isolator to protect sensitive quantum apparatus is the noise emitted in the backward direction, given by ${N_{{\rm{bw}}}}(0) = 1{\rm{/}}2 + ({\bar n_{{\rm{m}},1}} + {\bar n_{{\rm{m}},2}}){\rm{/}}2$. Here the noise is directly commensurate with the occupation of the mechanics which can be of hundreds of quanta even at cryogenic millikelvin temperatures, due to the low mechanical frequencies. This is a direct consequence of isolation without reflection, since it prevents fluctuations from either cavity to emerge in the backward direction. In order to preserve the commutation relations of the bosonic output modes, the fluctuations consequently have to originate from the mechanical modes. A practical low-noise design, therefore, requires a scheme to externally cool the mechanical modes, e.g. via sideband cooling using an additional auxiliary microwave mode.

**Fig. 4:**
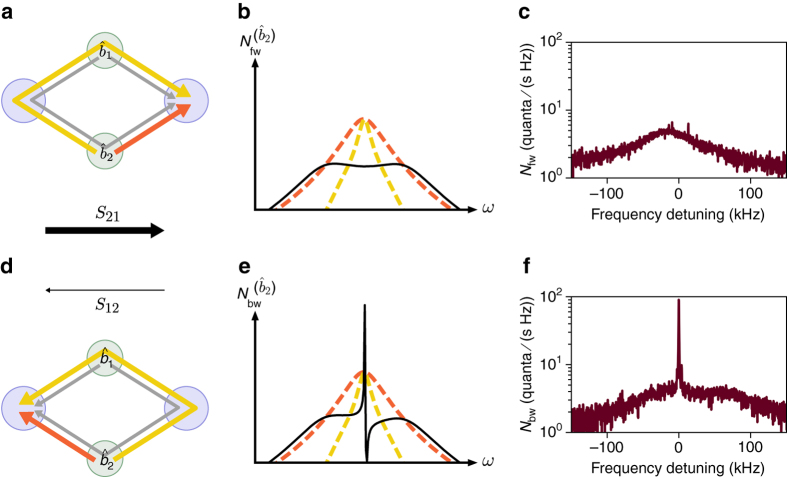
Asymmetric noise emission of the nonreciprocal circuit. The noise emission is mainly due to mechanical thermal noise, that is converted through two paths to the microwave modes. The resulting interference creates a different noise pattern in the forward **a**–**c** and the backward **d**–**f** directions when the circuit is tuned as an isolator from mode ${{\hat a}_1}$ to ${{\hat a}_2}$. **a**, **d** The two possible paths for the noise are shown for each mechanical mode. For ${\hat b_2}$, the direct path (*orange*) and the indirect path going through mode ${\hat b_1}$ (*yellow*) are highlighted (the corresponding paths for ${\hat b_1}$ are shown in *grey*). **b**, **e** Each path on its own would result in a wide noise spectrum that is equally divided between the two microwave cavities (*dashed yellow* and *orange lines*). When both paths are available, however, the noise interferes differently in each direction (*solid lines*). In the backward direction **e**, a sharp interference peak appears, of much larger amplitude than the broad base. The theoretical curves (on an arbitrary logarithmic scale) are shown for the symmetric case (Γ_m,1_ = Γ_m,2_) and for the single mode ${\hat b_2}$. Note that for the mode ${\hat b_1}$, the shape of the asymmetric peak in the backward noise would be the mirror image. **c**, **f** Measured output spectra of modes ${{\hat a}_2}$ (**c**) and ${{\hat a}_1}$ (**f**), calibrated to show the photon flux leaving the circuit. Because cross-damping provides extra cooling for the mode ${\hat b_1}$, the thermal noise of ${\hat b_2}$ is expected to dominate

The origin of this noise asymmetry can be understood as noise interference. The thermal fluctuations of one mechanical oscillator are converted to microwave noise in each cavity through two paths, illustrated in Fig. 4a, d: a direct (*orange*) and an indirect (*yellow*) link. Each pathway, on its own and with the same coupling strength, would result in symmetric noise that decreases in magnitude with increasing cooperativity. When both are present, however, the noise interferes with itself differently in each direction (Supplementary Note 3). In the forward direction, the noise interferes destructively (Fig. 4b) leading to low added noise, but in the backward direction, a sharp interference peak arises (Fig. 4e) with finite noise in the nonreciprocal bandwidth even in the high-cooperativity limit. In an intuitive picture, the circuit acts as a circulator that routes noise from the output port to the mechanical thermal bath and in turn the mechanical noise to the input port. We demonstrate experimentally the noise asymmetry by detecting the output spectra at each microwave mode while the device isolates the mode ${{\hat a}_1}$ from ${{\hat a}_2}$ by more than 25 dB (Fig. 4c, f). The cooperativities are here set to $({{\cal C}_{11}},{{\cal C}_{21}},{{\cal C}_{12}},{{\cal C}_{22}}) = (20.0,14.2,106,89)$ with a cross-damping $\Gamma _{{\rm{m,1}}}^{{\rm{(cross)}}} \approx 2\pi \cdot 2.6$ kHz, in order to optimize the circuit for a lower insertion loss and increase the noise visibility. As there is additional cooling from the off-resonant pump on mode ${\hat b_1}$, we expect noise from ${\hat b_2}$ to dominate.

### Quantum-limited circulator

**Fig. 5:**
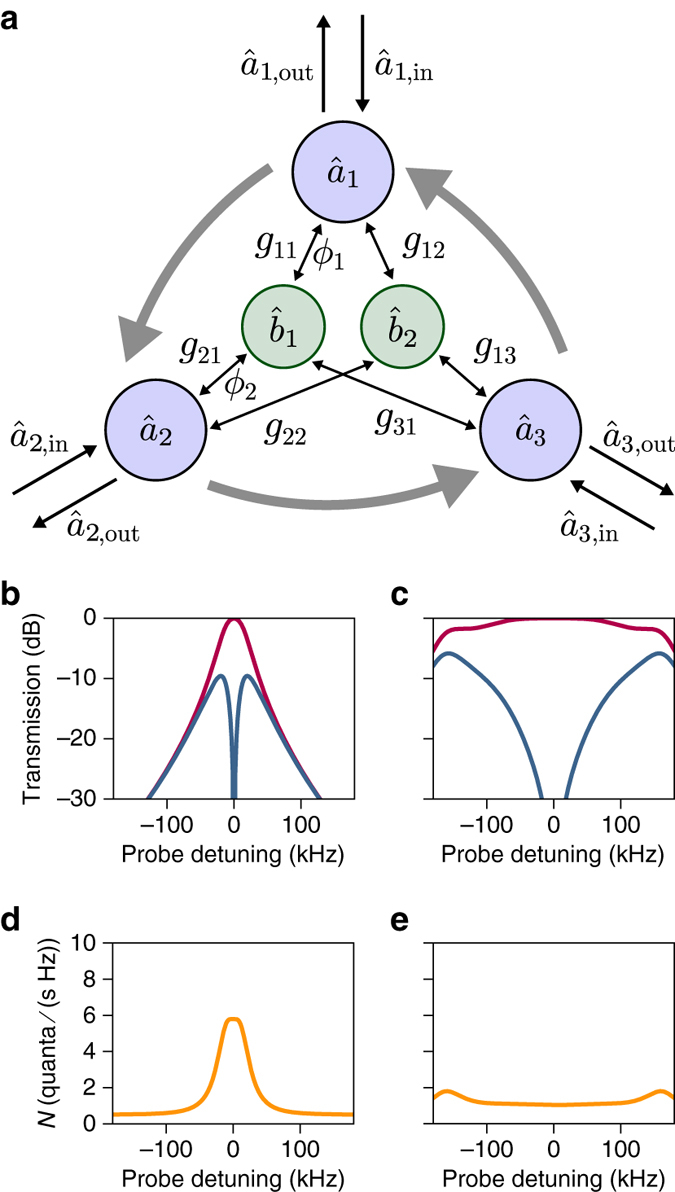
Proposal for a microwave optomechanical circulator. **a** With a third microwave mode ${{\hat a}_3}$ coupled to the same two mechanical oscillators, circulation can be achieved between the three microwave cavities. The circuit now involves two independent loops, with two phases *ϕ*_1_ and *ϕ*_2_ that can be tuned with the phases associated with *g*_21_ and *g*_11_, respectively. **b**, **c** The theoretical transmission in the circulating direction (counter-clockwise, in *red*) and the opposite direction (clockwise, in *blue*) are shown for the cooperativities ${\cal C} = 100$ (**b**) and ${\cal C} = 1000$ (**c**). The isolation bandwidth scales with ${\cal C}$ and is only limited by the energy decay rates of the microwave modes. Experimentally realistic parameters are chosen with overcoupled cavities of energy decay rates *κ*_1_ = *κ*_2_ = *κ*_3_ = 2*π* ⋅ 200 kHz and Γ_m,1_ = Γ_m,2_ = 2*π* ⋅ 100 Hz. **d**, **e** Noise emission spectra for the same two cooperativities (${\cal C} = 100$ (**d**) and ${\cal C} = 1000$ (**e**)), for ${\bar n_{{\rm{m}},1}} = {\bar n_{{\rm{m}},2}} = 800$. Note that for the circulator the noise is symmetric for all the cavities, and that it decreases with increasing cooperativity

There exists a way to circumvent the mechanical noise entirely: introducing one extra microwave mode ${{\hat a}_3}$, we can realize a circulator, where instead of mechanical fluctuations, the fluctuations from the third microwave mode emerge in the backward direction. The scheme is illustrated in Fig. 5a. As before, the two mechanical modes are used to create two interfering pathways, now between the three microwave cavities. Since there are now two independent loops, two phases matter; we choose the phases associated to the couplings *g*_11_ and *g*_21_ and set them respectively to *ϕ*_1_ = 2*π*/3 and *ϕ*_2_ = −2*π*/3. With the mechanical detunings set to ${\delta _i} = \frac{{\sqrt 3 }}{2}({\cal C} + \frac{1}{3}){\Gamma _{{\rm{m}},i}}$, the system then becomes a circulator that routes the input of port ${{\hat a}_1}$ to ${{\hat a}_2}$, ${{\hat a}_2}$ to ${{\hat a}_3}$ and so on (Supplementary Note 4). Critically and in contrast to above, counter-propagating signals are not dissipated in the mechanical oscillators, but directed to the other port, with two advantages. First, the bandwidth of nonreciprocity is not limited to the mechanical dissipation rate but instead increases with ${\cal C}$ until reaching the ultimate limit given by the cavity linewidth (Fig. 5b, c). Second, the mechanical noise emission is symmetrically spread between the three modes, and over the wide conversion bandwidth (Fig. 5d, e). In the large cooperativity limit, the nonreciprocal process becomes quantum limited, irrespective of the temperature of the mechanical thermal baths.

## Discussion

In conclusion, we described and experimentally demonstrated a new scheme for reconfigurable nonreciprocal transmission in the microwave domain using a superconducting optomechanical circuit. This scheme is based purely on optomechanical couplings, thus it alleviates the need for coherent microwave cavity–cavity (or direct phonon–phonon) interactions, and significantly facilitates the experimental realization, in contrast to recently used approaches of optomechanical nonreciprocity in the optical domain^37^. Nonreciprocity arises due to interference in the two mechanical modes, which mediate the microwave cavity–cavity coupling. This interference also manifests itself in the asymmetric noise output of the circuit. This scheme can be readily extended to implement quantum-limited phase-preserving and phase-sensitive directional amplifiers^45^. Moreover, an additional microwave mode enables quantum-limited microwave circulators on-chip with large bandwidth, limited only by the energy decay rate of the microwave modes. Finally, the presented scheme can be generalized to an array, and thus can form the basis to create topological phases of light and sound^46^ or topologically protected chiral amplifying states^47^ in arrays of electromechanical circuits, without requiring cavity–cavity or phonon–phonon mode hopping interactions.

### Data availability

The code and data used to produce the plots within this paper are available at http://dx.doi.org/10.5281/zenodo.816171. All other data used in this study are available from the corresponding authors upon reasonable request.

## Electronic supplementary material

Supplementary Information

Peer Review File
