# Supplementary material for: Nonreciprocal reconfigurable microwave optomechanical circuit
Source: Nat Commun. 2017 Sep 19;8:604. doi: 10.1038/s41467-017-00447-1 (PMC5605717; doi:10.1038/s41467-017-00447-1)
Supplement: Supplementary file 1 — Supplementary Information [file 41467_2017_447_MOESM1_ESM.pdf]

Description of Supplementary Files

File Name: Supplementary Information
Description: Supplementary Figure, Supplementary Notes and Supplementary References

File Name: Peer Review File

SUPPLEMENTARY NOTE 1: THEORETICAL BACKGROUND

In this note we derive the effective Hamiltonian relevant for our system, calculate the input-output scattering matrix for the electromagnetic modes and discuss the conditions for obtaining nonreciprocal microwave transmission.

We consider two mechanical degrees of freedom whose positions parametrically modulate the frequencies of two electromagnetic modes via radiation-pressure coupling [1]. The Hamiltonian describing this situation is given by ($\hbar = 1$)

$$\hat{H} = \sum_{i=1}^{2} \left(\omega_{c,i} \hat{a}_i^\dagger \hat{a}_i + \Omega_i \hat{b}_i^\dagger \hat{b}_i \right) + \hat{H}_{\rm int} + \hat{H}_{\rm drive}, \tag{1}$$

where $\hat{a}_1$ and $\hat{a}_2$ are the annihilation operators associated respectively with the two electromagnetic modes with frequencies $\omega_{c,1}$ and $\omega_{c,2}$, $\hat{b}_1$ and $\hat{b}_2$ are those for the two mechanical modes with respective mechanical frequencies Ω_1 and Ω_2, and $\hat{H}_{\rm drive}$ describes the electromagnetic pumps. Radiation-pressure coupling between the microwave and mechanical modes is described by the interaction Hamiltonian [1]

$$\hat{H}_{\rm int} = -\sum_{j=1}^{2} \sum_{k=1}^{2} g_{0,jk}\, \hat{a}_j^\dagger \hat{a}_j (\hat{b}_k + \hat{b}_k^\dagger), \tag{2}$$

with $g_{0,jk}$ the vacuum optomechanical coupling strength between electromagnetic mode j and mechanical mode k and where we neglect cross coupling terms $\propto \hat{a}_i^\dagger \hat{a}_j$, which is a good approximation for spectrally distinct modes $|\omega_{c,1} - \omega_{c,2}| \gg \Omega_i$ [2, 3].

In the experiment, both cavity modes are driven with two microwave tones each. These four tones are close to the lower mechanical sidebands, but the ones driving the mechanical sidebands at frequency Ω_1 are slightly detuned to the red, whereas the ones driving the sidebands at frequency Ω_2 are slightly detuned to the blue from the lower sideband. That is, the detuning of the four drives are $\Delta_{jk} = \omega_{jk} - \omega_{c,j}$ with $\Delta_{11} = \Delta_{21} = -\Omega_1 - \delta$ and $\Delta_{12} = \Delta_{22} = -\Omega_2 + \delta$.

We separate mean and fluctuations in the microwave fields and move to a frame rotating at the cavity frequencies

$$\hat{a}_j = e^{-i\omega_{c,j} t} \left((\delta \hat{a}_j) + \sum_{k=1}^{2} \alpha_{jk}\, e^{-i\Delta_{jk} t} \right) \tag{3}$$

where α_{jk} is the coherent state amplitude due to the microwave drive with detuning Δ_{jk} with $j, k = 1, 2$ and $(\delta \hat{a}_j)$ describe the fluctuations of the two microwave modes $j = 1, 2$. We then linearize the Hamiltonian by approximating

$$\hat{a}_j^\dagger \hat{a}_j \approx \left(\delta \hat{a}_j^\dagger \right) \left(\sum_{k=1}^{2} \alpha_{jk}\, e^{-i\Delta_{jk} t} \right) + \text{H.c.} \tag{4}$$

To obtain a time-independent Hamiltonian we will assume that the system is in the resolved-sideband limit with respect to both mechanical modes, i.e. $\Omega_1, \Omega_2 \gg \kappa_1, \kappa_2$, and that the two mechanical modes are well separated in frequency, i.e. $|\Omega_1 - \Omega_2| \gg \Gamma_{m,1}, \Gamma_{m,2}$. Moving into a rotating frame with respect to the free evolution of the microwave modes, and keeping only non-rotating terms, we obtain the effective Hamiltonian describing our system, which is given as equation (1) in the main manuscript,

$$\hat{H} = -\delta\, \hat{b}_1^\dagger \hat{b}_1 + \delta\, \hat{b}_2^\dagger \hat{b}_2 + g_{11}(\hat{a}_1 \hat{b}_1^\dagger + \hat{a}_1^\dagger \hat{b}_1) + g_{21}(\hat{a}_2 \hat{b}_1^\dagger + \hat{a}_2^\dagger \hat{b}_1) + g_{12}(\hat{a}_1 \hat{b}_2^\dagger + \hat{a}_1^\dagger \hat{b}_2) + g_{22}(e^{i\phi} \hat{a}_2 \hat{b}_2^\dagger + e^{-i\phi} \hat{a}_2^\dagger \hat{b}_2). \tag{5}$$

Here, $g_{jk} = g_{0,jk}|\alpha_{jk}|$ are the optomechanical coupling strengths enhanced by the mean intracavity photon numbers $n_{jk} = |\alpha_{jk}|^2$ due to the drive at frequency ω_{jk} and where we have renamed $(\delta \hat{a}_j) \to \hat{a}_j$ for notational convenience. Without loss of generality, the phase of all but one coupling constant g_{jk} can be chosen real. Here, we take all of them real and write out the phase ϕ explicitly which is varied in our experiment.

From the Hamiltonian (supplementary eq. (5)) we derive the equations of motion for our system which can be written in matrix form as [4, 5]

$$\dot{\mathbf{u}} = M\, \mathbf{u} + L\, \mathbf{u}_{\rm in} \tag{6}$$

with $\mathbf{u} = (\hat{a}_1, \hat{a}_2, \hat{b}_1, \hat{b}_2)^T$, $\mathbf{u}_{\rm in} = (\hat{a}_{1,\rm in}, \hat{a}_{2,\rm in}, \hat{a}_{1,\rm in}^{(0)}, \hat{a}_{2,\rm in}^{(0)}, \hat{b}_{1,\rm in}, \hat{b}_{2,\rm in})^T$ and $\mathbf{u}_{\rm out} = (\hat{a}_{1,\rm out}, \hat{a}_{2,\rm out}, \hat{a}_{1,\rm out}^{(0)}, \hat{a}_{2,\rm out}^{(0)}, \hat{b}_{1,\rm out}, \hat{b}_{2,\rm out})^T$, where $\hat{a}_{i,\rm in/out}$ are the input-output modes of the external microwave feedline and $\hat{a}_{i,\rm in/out}^{(0)}$ are those corresponding to internal dissipation.

The matrix M reads

$$M = \begin{pmatrix} -\frac{\kappa_1}{2} & 0 & -ig_{11} & -ig_{12} \\ 0 & -\frac{\kappa_2}{2} & -ig_{21} & -ig_{22}e^{-i\phi} \\ -ig_{11} & -ig_{21} & +i\delta - \frac{\Gamma_{\rm m,1}}{2} & 0 \\ -ig_{12} & -ig_{22}e^{+i\phi} & 0 & -i\delta - \frac{\Gamma_{\rm m,2}}{2} \end{pmatrix} \tag{7}$$

where the cavity dissipation rates are the sum of external and internal dissipation rates, i.e. $\kappa_1 = \kappa_{\text{ex},1} + \kappa_{0,1}$ and $\kappa_2 = \kappa_{\text{ex},2} + \kappa_{0,2}$, and the matrix L reads

$$L = \begin{pmatrix} \sqrt{\kappa_{\text{ex},1}} & 0 & \sqrt{\kappa_{0,1}} & 0 & 0 & 0 \\ 0 & \sqrt{\kappa_{\text{ex},2}} & 0 & \sqrt{\kappa_{0,2}} & 0 & 0 \\ 0 & 0 & 0 & 0 & \sqrt{\Gamma_{\text{m},1}} & 0 \\ 0 & 0 & 0 & 0 & 0 & \sqrt{\Gamma_{\text{m},2}} \end{pmatrix}. \tag{8}$$

Using the input-output relations for a one-sided cavity [4, 5]

$$\mathbf{u}_{\text{out}} = \mathbf{u}_{\text{in}} - L^T\, \mathbf{u} \tag{9}$$

we can solve the input-output problem in the Fourier domain

$$\mathbf{u}_{\text{out}}(\omega) = S(\omega)\, \mathbf{u}_{\text{in}}(\omega) \tag{10}$$

with the scattering matrix

$$S(\omega) = \mathbb{1}_{6\times 6} + L^T\, \left[+i\omega \mathbb{1}_{4\times 4} + M\right]^{-1} L. \tag{11}$$

Eliminating the mechanical degrees of freedom from the equations of motion (supplementary eq. (6)) we obtain

$$\begin{pmatrix} \frac{\kappa_1}{2} - i\omega + g_{11}^2\chi_1(\omega) + g_{12}^2\chi_2(\omega) & g_{11}\chi_1(\omega)g_{21} + g_{12}\chi_2(\omega)g_{22}e^{+i\phi} \\ g_{11}\chi_1(\omega)g_{21} + g_{12}\chi_2(\omega)g_{22}e^{-i\phi} & \frac{\kappa_2}{2} - i\omega + g_{21}^2\chi_1(\omega) + g_{22}^2\chi_2(\omega) \end{pmatrix} \begin{pmatrix} \hat{a}_1 \\ \hat{a}_2 \end{pmatrix}$$
$$= \begin{pmatrix} \sqrt{\kappa_{\text{ex},1}}\hat{a}_{\text{in},1} + \sqrt{\kappa_{0,1}}\hat{a}^{(0)}_{\text{in},1} - ig_{11}\chi_1(\omega)\sqrt{\Gamma_{\text{m},1}}\hat{b}_{1,\text{in}} - ig_{12}\chi_2(\omega)\sqrt{\Gamma_{\text{m},2}}\hat{b}_{2,\text{in}} \\ \sqrt{\kappa_{\text{ex},2}}\hat{a}_{\text{in},2} + \sqrt{\kappa_{0,2}}\hat{a}^{(0)}_{\text{in},2} - ig_{21}\chi_1(\omega)\sqrt{\Gamma_{\text{m},1}}\hat{b}_{1,\text{in}} - ig_{22}\chi_2(\omega)e^{-i\phi}\sqrt{\Gamma_{\text{m},2}}\hat{b}_{2,\text{in}} \end{pmatrix} \tag{12}$$

where we introduced the mechanical susceptibilities $\chi_1^{-1}(\omega) = \Gamma_{\text{m},1}/2 - i\,(\delta + \omega)$ and $\chi_2^{-1}(\omega) = \Gamma_{\text{m},2}/2 + i\,(\delta - \omega)$. Inverting the matrix in supplementary eq. (12) and exploiting the input-output relation (supplementary eq. (9)), we obtain equation (3) of the main manuscript

$$\frac{S_{12}(\omega)}{S_{21}(\omega)} = \frac{g_{11}\chi_1(\omega)g_{21} + g_{12}\chi_2(\omega)g_{22}e^{+i\phi}}{g_{11}\chi_1(\omega)g_{21} + g_{12}\chi_2(\omega)g_{22}e^{-i\phi}}. \tag{13}$$

Note that the expressions in the nominator and denominator in supplementary eq. (13) are equal to the matrix elements coupling the two electromagnetic modes in supplementary eq. (12) which are the sum of the two (complex) amplitudes for the two dissipative optomechanical pathways. Supplementary eq. (13) is used to generate Fig. 3 E of the main text, with all the parameters $(\Gamma_{\text{m},j}, g_{ij})$ independently measured.

For identical mechanical decay rates $\Gamma_{\text{m},1} = \Gamma_{\text{m},2} = \Gamma_{\text{m}}$ and identical cooperativities $\mathcal{C} = \mathcal{C}_{ij} = \frac{4g_{ij}^2}{\kappa_i \Gamma_{m,j}}$ we find that the transmission $2 \to 1$ vanishes on resonance $\omega = 0$, i.e. $S_{12} = 0$, if

$$\frac{\Gamma_{\text{m}}}{2\delta} = \tan\frac{\phi}{2}. \tag{14}$$

For a given δ, maximal transmission in the opposite direction $1 \to 2$ is then obtained for $\mathcal{C} = \frac{1}{2} + \frac{2\delta^2}{\Gamma_{\text{m}}^2}$ and given by

$$|S_{21}|^2_{\text{max}} = \frac{\kappa_{\text{ex},1}\kappa_{\text{ex},2}}{\kappa_1\kappa_2}\frac{4\delta^2}{\Gamma_{\text{m}}^2 + 4\delta^2} = \frac{\kappa_{\text{ex},1}\kappa_{\text{ex},2}}{\kappa_1\kappa_2}\left(1 - \frac{1}{2\mathcal{C}}\right). \tag{15}$$

We see that for $\delta \gg \Gamma_{\text{m}}$ the optimal cooperativity $\mathcal{C} \to \infty$ and $|S_{21}(0)|^2 \to 1$. Thus, we see that in this limit the electromagnetic scattering matrix of our system becomes that of an ideal isolator, i.e. $S_{11} = S_{12} = S_{22} = 0$ and $|S_{21}| = 1$.

The full scattering matrix S_{ij} of supplementary eq. (11) is used in Supplementary Fig. 1 to show optimal transmission in each direction for the symmetric case, with different values of the cooperativity. As the cooperativity increases, the overall bandwidth of conversion increases to Γ_{eff}, but the nonreciprocal bandwidth stays constant. This can be seen in the ratio $S_{12}(\omega)/S_{21}(\omega)$ in supplementary eq. (13) that depends only on the bare mechanical susceptibilities $\chi_1(\omega)$ and $\chi_2(\omega)$.

For unequal decay rates $\Gamma_{\text{m},1} \neq \Gamma_{\text{m},2}$, but equal effective decay rates of the mechanical modes $\Gamma_{\text{eff},j} = \Gamma_{\text{m},j}(1 + \mathcal{C}_{1j} + \mathcal{C}_{2j})$, nonreciprocity is obtained for $\frac{\Gamma_+}{2\delta} = \tan\frac{\phi}{2}$ off-resonance at a frequency $\omega = \frac{\Gamma_+\Gamma_-}{4\delta}$ where $\Gamma_\pm = \frac{1}{2}\left(\Gamma_{\text{m},1} \pm \Gamma_{\text{m},2}\right)$. For unequal decay rates $\Gamma_{\text{m},1} \neq \Gamma_{\text{m},2}$, but matched cooperativities $\mathcal{C}_{jk} = \mathcal{C}$, we find nonreciprocity for $\frac{\Gamma_+}{2\delta} = \tan\frac{\phi}{2}$, but at $\omega = -\frac{\Gamma_-\delta}{\Gamma_+}$.

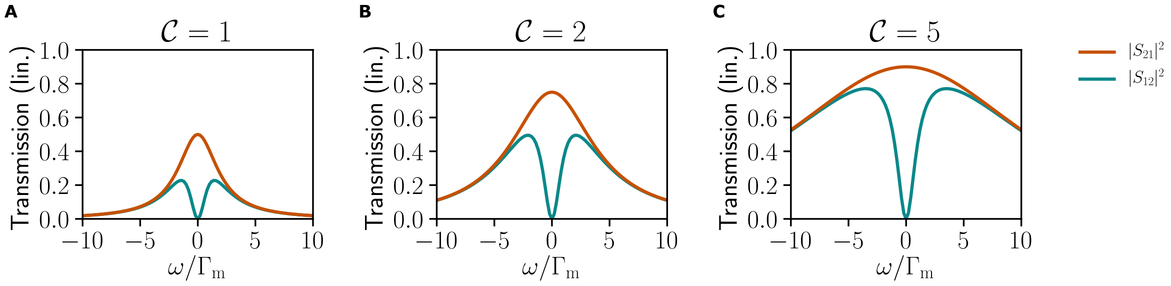

Supplementary Fig. 1. Microwave transmission of the nonreciprocal electromechanical device in each direction for different values of the cooperativity $\mathcal{C}$, derived from supplementary eq. (11) for the case of symmetric mechanical modes ($\Gamma_{\mathrm{m},1} = \Gamma_{\mathrm{m},2} = \Gamma_\mathrm{m}$). The detuning δ and the phase ϕ are set for maximal transmission according to supplementary eq. (15). As the cooperativity is increased, the overall bandwidth of the frequency conversion increases to Γ_eff, however the bandwidth of nonreciprocal transmission stays constant and is on the order of the intrinsic mechanical damping rate Γ_m. This illustrates the fact that the intrinsic dissipation of the mechanical oscillator is the underlying resource for the nonreciprocity.

SUPPLEMENTARY NOTE 2: NOISE ANALYSIS OF THE DEVICE

In this note we analyse the noise properties of the nonreciprocal electromechanical device. We assume the bosonic input noise operators obey

$$\langle \hat{a}_{1,\mathrm{in}}(t)\hat{a}^\dagger_{1,\mathrm{in}}(t')\rangle = \delta(t-t') \tag{16}$$
$$\langle \hat{a}_{2,\mathrm{in}}(t)\hat{a}^\dagger_{2,\mathrm{in}}(t')\rangle = \delta(t-t') \tag{17}$$
$$\langle \hat{a}^{(0)}_{1,\mathrm{in}}(t)\hat{a}^{(0)\dagger}_{1,\mathrm{in}}(t')\rangle = \delta(t-t') \tag{18}$$
$$\langle \hat{a}^{(0)}_{2,\mathrm{in}}(t)\hat{a}^{(0)\dagger}_{2,\mathrm{in}}(t')\rangle = \delta(t-t') \tag{19}$$
$$\langle \hat{b}_{1,\mathrm{in}}(t)\hat{b}^\dagger_{1,\mathrm{in}}(t')\rangle = (\bar{n}_{\mathrm{m},1}+1)\delta(t-t') \tag{20}$$
$$\langle \hat{b}_{2,\mathrm{in}}(t)\hat{b}^\dagger_{2,\mathrm{in}}(t')\rangle = (\bar{n}_{\mathrm{m},2}+1)\delta(t-t'), \tag{21}$$

i.e. the baths of the microwave modes are assumed to be at zero temperature whereas the mechanical modes have a finite thermal occupation $\bar{n}_{\mathrm{m},1}$ and $\bar{n}_{\mathrm{m},2}$, respectively.

The symmetrised output noise spectra [5] are determined by the scattering matrix of the device (supplementary eq. (11)) as well as the noise properties of the microwave and mechanical baths (supplementary eq. (16) to (21)). Explicitly, we find that the cavity output spectra are given by

$$\begin{aligned}\bar{S}_{1,\mathrm{out}}(\omega) &= \frac{1}{2}\int_{-\infty}^{\infty} dt\, e^{i\omega t}\langle \hat{a}^\dagger_{1,\mathrm{out}}(t)\hat{a}_{1,\mathrm{out}}(0) + \hat{a}_{1,\mathrm{out}}(0)\hat{a}^\dagger_{1,\mathrm{out}}(t)\rangle \\ &= \tfrac{1}{2}\left[|S_{11}(-\omega)|^2 + |S_{12}(-\omega)|^2 + |S_{13}(-\omega)|^2 + |S_{14}(-\omega)|^2\right] + |S_{15}(-\omega)|^2(\bar{n}_{\mathrm{m},1}+\tfrac{1}{2}) + |S_{16}(-\omega)|^2(\bar{n}_{\mathrm{m},2}+\tfrac{1}{2})\end{aligned} \tag{22}$$

and

$$\begin{aligned}\bar{S}_{2,\mathrm{out}}(\omega) &= \frac{1}{2}\int_{-\infty}^{\infty} dt\, e^{i\omega t}\langle \hat{a}^\dagger_{2,\mathrm{out}}(t)\hat{a}_{2,\mathrm{out}}(0) + \hat{a}_{2,\mathrm{out}}(0)\hat{a}^\dagger_{2,\mathrm{out}}(t)\rangle \\ &= \tfrac{1}{2}\left[|S_{21}(-\omega)|^2 + |S_{22}(-\omega)|^2 + |S_{23}(-\omega)|^2 + |S_{24}(-\omega)|^2\right] + |S_{25}(-\omega)|^2(\bar{n}_{\mathrm{m},1}+\tfrac{1}{2}) + |S_{26}(-\omega)|^2(\bar{n}_{\mathrm{m},2}+\tfrac{1}{2}).\end{aligned} \tag{23}$$

In the limit of overcoupled cavities $\kappa_{\mathrm{ex},i} \approx \kappa_i$ and for the optimal phase ϕ and detuning δ, the noise emitted in the backward direction $2 \to 1$ on resonance $\omega = 0$ is

$$\begin{aligned}N_\mathrm{bw} = \bar{S}_{1,\mathrm{out}}(0) &= |S_{11}|^2 \times \frac{1}{2} + |S_{12}|^2 \times \frac{1}{2} + |S_{15}|^2 \times \left(\bar{n}_{\mathrm{m},1} + \frac{1}{2}\right) + |S_{16}|^2 \times \left(\bar{n}_{\mathrm{m},2} + \frac{1}{2}\right) \\ &= 0 \times \frac{1}{2} + 0 \times \frac{1}{2} + \frac{1}{2} \times \left(\bar{n}_{\mathrm{m},1} + \frac{1}{2}\right) + \frac{1}{2} \times \left(\bar{n}_{\mathrm{m},2} + \frac{1}{2}\right) \\ &= \frac{1}{2} + \frac{\bar{n}_{\mathrm{m},1} + \bar{n}_{\mathrm{m},2}}{2},\end{aligned} \tag{24}$$

i.e. in the backward direction the noise of the device is dominated by the noise emitted by the mechanical oscillators.

The noise emitted in the forward direction $1 \to 2$ on resonance $\omega = 0$ is

$$\begin{aligned} N_{\text{fw}} = \bar{S}_{2,\text{out}}(0) &= |S_{21}|^2 \times \frac{1}{2} + |S_{22}|^2 \times \frac{1}{2} + |S_{25}|^2 \times \left(\bar{n}_{\text{m},1} + \frac{1}{2}\right) + |S_{26}|^2 \times \left(\bar{n}_{\text{m},2} + \frac{1}{2}\right) \\ &= \left(1 - \frac{1}{2\mathcal{C}}\right) \times \frac{1}{2} + 0 \times \frac{1}{2} + \frac{1}{4\mathcal{C}} \times \left(\bar{n}_{\text{m},1} + \frac{1}{2}\right) + \frac{1}{4\mathcal{C}} \times \left(\bar{n}_{\text{m},2} + \frac{1}{2}\right) \\ &= \frac{1}{2} + \frac{\bar{n}_{\text{m},1} + \bar{n}_{\text{m},2}}{4\mathcal{C}}, \end{aligned} \tag{25}$$

i.e. in the forward direction the noise contribution from the mechanical oscillators vanishes at large cooperativity $\mathcal{C} \gg 1$. Therefore, intriguingly, the noise emitted on resonance by the nonreciprocal device is not symmetric in the forward and backward directions.

SUPPLEMENTARY NOTE 3: NOISE INTERFERENCE AS ORIGIN OF ASYMMETRIC NOISE EMISSION

In the previous note we concluded that the circuit emits more noise in the backward direction as compared to the forward direction. This is also corroborated by the experimental data, shown in Fig. 4 in the main text. In the following, in order to understand the different noise performance in the forward and backward direction, we consider two different points of view. First, we derive the scattering amplitude from one mechanical resonator to one cavity, eliminating the other two modes. In this picture, the imbalance can be understood as an interference of the two paths the noise can take in the circuit, analogously to the interference in the microwave transmission. Second, we eliminate the mechanical resonators, but taking their input noise into account. This leads to the same scattering matrix for the microwaves as discussed in the main text, but the mechanical noise appears as additional, effective noise input operators for the cavities. In the second formulation we can therefore use our knowledge of the microwave scattering matrix to deduce properties of the noise scattering.

Let us first consider the scattering from a mechanical resonator to cavities 1 and 2. Since in the experiment mechanical resonator 1 is strongly cross-damped due to off-resonant couplings, the noise emitted stems almost exclusively from resonator 2. If we are interested in the noise scattering from mechanical resonator 2 to cavity 2, we can eliminate the other two modes and drop their input noise. In frequency space, their equations of motion are

$$\begin{pmatrix} \chi_{c,1}^{-1}(\omega) & -ig_{11} \\ -ig_{11}^* & \chi_1^{-1}(\omega) \end{pmatrix} \begin{pmatrix} \hat{a}_1 \\ \hat{b}_1 \end{pmatrix} = \begin{pmatrix} ig_{12}\hat{b}_2 \\ ig_{21}^*\hat{a}_2 \end{pmatrix} + \text{noises}. \tag{26}$$

We drop the noise terms and solve for $\hat{a}_1, \hat{b}_1$

$$\begin{aligned} \begin{pmatrix} \hat{a}_1(\omega) \\ \hat{b}_1(\omega) \end{pmatrix} &= \frac{1}{\chi_1^{-1}(\omega)\chi_{c,1}^{-1}(\omega) + |g_{11}|^2} \begin{pmatrix} \chi_1^{-1}(\omega) & ig_{11} \\ ig_{11}^* & \chi_{c,1}^{-1}(\omega) \end{pmatrix} \begin{pmatrix} ig_{12}\hat{b}_2(\omega) \\ ig_{21}^*\hat{a}_2(\omega) \end{pmatrix} \\ &\equiv \chi_{\hat{a}_1\hat{b}_1}(\omega) \begin{pmatrix} ig_{12} & \\ & ig_{21}^* \end{pmatrix} \begin{pmatrix} \hat{b}_2(\omega) \\ \hat{a}_2(\omega) \end{pmatrix}, \end{aligned} \tag{27}$$

where we defined the cavity susceptibility $\chi_{c,i}^{-1}(\omega) = \kappa_i/2 - i\omega$ and the susceptibility of the coupled system of modes $\hat{a}_1, \hat{b}_1$, $\chi_{\hat{a}_1\hat{b}_1}(\omega)$. We turn to the other two modes, the ones that we are actually interested in. For those, we have a similar equation, which can be obtained from interchanging $1 \leftrightarrow 2$

$$\begin{pmatrix} \chi_2^{-1}(\omega) & -ig_{22}^* \\ -ig_{22} & \chi_{c,2}^{-1}(\omega) \end{pmatrix} \begin{pmatrix} \hat{b}_2(\omega) \\ \hat{a}_2(\omega) \end{pmatrix} = \begin{pmatrix} ig_{12}^* & \\ & ig_{21} \end{pmatrix} \begin{pmatrix} \hat{a}_1(\omega) \\ \hat{b}_1(\omega) \end{pmatrix} + \begin{pmatrix} \sqrt{\Gamma_{\text{m},2}}\hat{b}_{2,\text{in}}(\omega) \\ \sqrt{\kappa_2}\hat{a}_{2,\text{in}}(\omega) \end{pmatrix}. \tag{28}$$

Eliminating the modes $\hat{a}_1, \hat{b}_1$ with supplementary eq. (27), we arrive at

$$\begin{aligned} \begin{pmatrix} \sqrt{\Gamma_{\text{m},2}}\hat{b}_{2,\text{in}}(\omega) \\ \sqrt{\kappa_2}\hat{a}_{2,\text{in}}(\omega) \end{pmatrix} &= \left[\begin{pmatrix} \chi_2^{-1}(\omega) & -ig_{22}^* \\ -ig_{22} & \chi_{c,2}^{-1}(\omega) \end{pmatrix} - \frac{\begin{pmatrix} ig_{12}^* & \\ & ig_{21} \end{pmatrix} \begin{pmatrix} \chi_1^{-1}(\omega) & ig_{11} \\ ig_{11}^* & \chi_{c,1}^{-1}(\omega) \end{pmatrix} \begin{pmatrix} ig_{12} & \\ & ig_{21}^* \end{pmatrix}}{\chi_1^{-1}(\omega)\chi_{c,1}^{-1}(\omega) + |g_{11}|^2} \right] \begin{pmatrix} \hat{b}_2(\omega) \\ \hat{a}_2(\omega) \end{pmatrix} \\ &\equiv \left[\chi_{\hat{b}_2\hat{a}_2}^{-1}(\omega) - \begin{pmatrix} ig_{12}^* & \\ & ig_{21} \end{pmatrix} \chi_{\hat{a}_1\hat{b}_1}(\omega) \begin{pmatrix} ig_{12} & \\ & ig_{21}^* \end{pmatrix} \right] \begin{pmatrix} \hat{b}_2(\omega) \\ \hat{a}_2(\omega) \end{pmatrix}. \end{aligned} \tag{29}$$

In the second line, we have formulated the equation in terms of the susceptibilities of the two subsystems $(\hat{a}_1, \hat{b}_1)$ and $(\hat{a}_2, \hat{b}_2)$. This equation is a bit complicated, but we note that the coupling between $\hat{a}_2$ and $\hat{b}_2$ is

$$iT_{\hat{a}_2\hat{b}_2}(\omega) = -ig_{22}\left[1 - e^{-i\phi_p}\frac{\mathcal{C}_{12}\mathcal{C}_{21}/(\mathcal{C}_{22}\mathcal{C}_{11})}{1 + \left(\chi_{c,1}(\omega)\chi_1(\omega)|g_{11}|^2\right)^{-1}}\right]. \tag{30}$$

Analogously, changing the indices referring to the cavity, we obtain the coupling between $\hat{a}_1$ and $\hat{b}_2$

$$iT_{\hat{a}_1\hat{b}_2}(\omega) = -ig_{12}\left[1 - e^{+i\phi_p}\frac{\mathcal{C}_{11}\mathcal{C}_{22}/(\mathcal{C}_{12}\mathcal{C}_{21})}{1 + \left(\chi_{c,2}(\omega)\chi_1(\omega)|g_{21}|^2\right)^{-1}}\right], \tag{31}$$

The coupling phase ϕ_p appears as the relative phase between indirect and direct coupling path, as for the microwave signal transmission. Equations (30) and (31) demonstrate that the transmission of noise from the mechanical resonators to the microwave cavities is subject to interference, which ultimately leads to the difference in noise emitted in the forward versus the backward direction.

In a second picture, we can also understand the mechanical noise interference in terms of the nonreciprocity in the scattering matrix for the microwave modes. In order to do so, we solve the equations of motion for the mechanical resonators (given in supplementary eq. (6)), which leads to

$$\hat{b}_j(\omega) = \chi_j(\omega)\left[i\sum_i g_{ij}^*\hat{a}_i(\omega) + \sqrt{\Gamma_{\mathrm{m},j}}\hat{b}_{j,\mathrm{in}}(\omega)\right]. \tag{32}$$

We obtain equations that only relate the cavities

$$\begin{pmatrix}\chi_{c,1}^{-1}(\omega) + iT_{11}(\omega) & iT_{12}(\omega)\\ iT_{21}(\omega) & \chi_{c,2}^{-1}(\omega) + iT_{22}(\omega)\end{pmatrix}\begin{pmatrix}\hat{a}_1(\omega)\\ \hat{a}_2(\omega)\end{pmatrix} = i\begin{pmatrix}g_{11} & g_{12}\\ g_{21} & g_{22}\end{pmatrix}\begin{pmatrix}\sqrt{\Gamma_{\mathrm{m},1}}\chi_1(\omega)\hat{b}_{1,\mathrm{in}}(\omega)\\ \sqrt{\Gamma_{\mathrm{m},2}}\chi_2(\omega)\hat{b}_{2,\mathrm{in}}(\omega)\end{pmatrix} + \begin{pmatrix}\sqrt{\kappa_1}\hat{a}_{1,\mathrm{in}}(\omega)\\ \sqrt{\kappa_2}\hat{a}_{2,\mathrm{in}}(\omega)\end{pmatrix}, \tag{33}$$

where

$$iT_{ij}(\omega) \equiv -i\sum_k \chi_k(\omega) g_{ik} g_{jk}^*. \tag{34}$$

We can think of mechanical noise as coloured and correlated noise in the optical inputs. That is, consider the replacement

$$\begin{pmatrix}\sqrt{\kappa_1}\hat{c}_{1,\mathrm{in}}(\omega)\\ \sqrt{\kappa_2}\hat{c}_{2,\mathrm{in}}(\omega)\end{pmatrix} \equiv i\begin{pmatrix}g_{11} & g_{12}\\ g_{21} & g_{22}\end{pmatrix}\begin{pmatrix}\sqrt{\Gamma_{\mathrm{m},1}}\chi_1(\omega)\hat{b}_{1,\mathrm{in}}(\omega)\\ \sqrt{\Gamma_{\mathrm{m},2}}\chi_2(\omega)\hat{b}_{2,\mathrm{in}}(\omega)\end{pmatrix}. \tag{35}$$

The effective noise $\hat{c}_{i,\mathrm{in}}$ is both coloured $\langle\hat{c}_{1,\mathrm{in}}^\dagger(\omega)\hat{c}_{1,\mathrm{in}}(\omega')\rangle \neq \delta(\omega+\omega')\bar{n}_{1,\mathrm{eff}}$ and correlated $\langle\hat{c}_{1,\mathrm{in}}^\dagger(\omega)\hat{c}_{2,\mathrm{in}}(\omega')\rangle \neq 0$.

Using the input-output relation $\hat{a}_{\mathrm{out}} = \hat{a}_{\mathrm{in}} - \sqrt{\kappa}\hat{a}$, the cavity output is given by

$$\begin{pmatrix}\hat{a}_{1,\mathrm{out}}(\omega)\\ \hat{a}_{2,\mathrm{out}}(\omega)\end{pmatrix} = S(\omega)\begin{pmatrix}\hat{a}_{1,\mathrm{in}}(\omega)\\ \hat{a}_{2,\mathrm{in}}(\omega)\end{pmatrix} + [S(\omega) - \mathbb{1}_2]\begin{pmatrix}\hat{c}_{1,\mathrm{in}}(\omega)\\ \hat{c}_{2,\mathrm{in}}(\omega)\end{pmatrix}, \tag{36}$$

where in the last step we have identified the 2-by-2 optical scattering matrix $S(\omega)$ that relates the cavity inputs to the outputs $\hat{a}_{i,\mathrm{out}}(\omega) = \sum_j S_{ij}(\omega)\hat{a}_{j,\mathrm{in}}(\omega)$. The fact that supplementary eq. (36) contains mechanical noise as well, but can be written entirely in terms of the optical scattering matrix constitutes the central result here. Since the two effective input noises $\hat{c}_{i,\mathrm{in}}$ are coloured and correlated, they can interfere.

Most importantly, we can consider what happens when the circuit is impedance matched to the signal and perfectly isolating. We choose the detunings $\delta_1 = \Gamma_{\mathrm{m},1}\delta/2, \delta_2 = -\Gamma_{\mathrm{m},2}\delta/2$, for some dimensionless parameter δ. For simplicity, let us choose all cooperativities to be equal $\mathcal{C} = \mathcal{C}_{ij}$. For $\delta^2 = 2\mathcal{C} - 1$ (impedance matching), the optical scattering matrix of the isolator is (up to some irrelevant phase)

$$S(0) = \begin{pmatrix}0 & 0\\ \sqrt{1 - 1/(2\mathcal{C})} & 0\end{pmatrix} \equiv T\begin{pmatrix}0 & 0\\ 1 & 0\end{pmatrix}. \tag{37}$$

The cavity output on resonance is

$$\begin{pmatrix}\hat{a}_{1,\mathrm{out}}\\ \hat{a}_{2,\mathrm{out}}\end{pmatrix} = T\begin{pmatrix}0\\ \hat{a}_{1,\mathrm{in}}\end{pmatrix} - \frac{i}{\sqrt{2}}\mathcal{C}\begin{pmatrix}e^{i\phi_p} & 1\\ 1 - Te^{i\phi_p} & 1 - T\end{pmatrix}\begin{pmatrix}\hat{b}_{1,\mathrm{in}}(0)\\ \hat{b}_{2,\mathrm{in}}(0)\end{pmatrix}. \tag{38}$$

As $\mathcal{C} \to \infty$, $T \to 1$ and $\phi_p = \arccos(1 - 1/\mathcal{C}) \to 0$, such that the second cavity does not receive any noise, which is due to an interference of $\hat{c}_{1,\mathrm{in}}$ with $\hat{c}_{2,\mathrm{in}}$. In the backward direction, no interference can take place, since cavity 2 is isolated from cavity 1. As a consequence, the number of noise quanta emerging from cavity 1 on resonance is $N_{\mathrm{bw}} = (\bar{n}_{\mathrm{m},1} + \bar{n}_{\mathrm{m},2} + 1)/2$.

SUPPLEMENTARY NOTE 4: OPTOMECHANICAL CIRCULATOR

In this note we present a scheme for a circulator based on the same principles as the isolator previously discussed. The scheme naturally overcomes the shortcomings of the isolator, becoming wideband and quantum limited in the high cooperativity limit.

We consider three microwave modes (described by their annihilation operators $\hat{a}_1$, $\hat{a}_2$, $\hat{a}_3$) with resonance frequencies $\omega_{c,1}$, $\omega_{c,2}$, $\omega_{c,3}$ and dissipation rates κ_1, κ_2, κ_3. These three microwave modes are coupled to two mechanical modes (described by the annihilation operators $\hat{b}_1$, $\hat{b}_2$) with resonance frequencies Ω_1, Ω_2 and dissipation rates $\Gamma_{\mathrm{m},1}$ and $\Gamma_{\mathrm{m},2}$. The optomechanical coupling strengths g_{ij} are taken to be real and we define three phases ϕ_1, ϕ_2 and ϕ_3 associated respectively to the couplings g_{11}, g_{21} and g_{31}. The three cavities are driven with two microwave tones each. These six tones are close to the lower motional sidebands, with detunings of $\Delta_{11} = \Delta_{21} = \Delta_{31} = -\Omega_1 + \delta_1$ and $\Delta_{12} = \Delta_{22} = \Delta_{32} = -\Omega_2 + \delta_2$. The cooperativities are set to be equal for all couplings, with $\mathcal{C} = \mathcal{C}_{ij} = \frac{4g_{ij}}{\kappa_i \Gamma_j}$.

The linearised Hamiltonian that describes the system, in a frame rotating with the cavity frequencies and keeping only time-constant terms is given by

$$\begin{aligned}\hat{H} =& \delta_1 \hat{b}_1^\dagger \hat{b}_1 + \delta_2 \hat{b}_2^\dagger \hat{b}_2 \\ &+ g_{11}(\hat{a}_1^\dagger \hat{b}_1 e^{i\phi_1} + \hat{a}_1 \hat{b}_1^\dagger e^{-i\phi_1}) + g_{12}(\hat{a}_1^\dagger \hat{b}_2 + \hat{a}_1 \hat{b}_2^\dagger) \\ &+ g_{21}(\hat{a}_2^\dagger \hat{b}_1 e^{i\phi_2} + \hat{a}_2 \hat{b}_1^\dagger e^{-i\phi_2}) + g_{22}(\hat{a}_2^\dagger \hat{b}_2 + \hat{a}_2 \hat{b}_2^\dagger) \\ &+ g_{31}(\hat{a}_3^\dagger \hat{b}_1 e^{i\phi_3} + \hat{a}_3 \hat{b}_1^\dagger e^{-i\phi_3}) + g_{32}(\hat{a}_3^\dagger \hat{b}_2 + \hat{a}_3 \hat{b}_2^\dagger).\end{aligned} \tag{39}$$

From this Hamiltonian, we derive the equations of motion for our system in the matrix form (as in supplementary eq. (6)) with $\mathbf{u} = (\hat{a}_1, \hat{a}_2, \hat{a}_3, \hat{b}_1, \hat{b}_2)^T$, $\mathbf{u}_{\mathrm{in}} = (\hat{a}_{1,\mathrm{in}}, \hat{a}_{2,\mathrm{in}}, \hat{a}_{3,\mathrm{in}}, \hat{a}^{(0)}_{1,\mathrm{in}}, \hat{a}^{(0)}_{2,\mathrm{in}}, \hat{a}^{(0)}_{3,\mathrm{in}}, \hat{b}_{1,\mathrm{in}}, \hat{b}_{2,\mathrm{in}})^T$ and $\mathbf{u}_{\mathrm{out}} = (\hat{a}_{1,\mathrm{out}}, \hat{a}_{2,\mathrm{out}}, \hat{a}_{3,\mathrm{out}}, \hat{a}^{(0)}_{1,\mathrm{out}}, \hat{a}^{(0)}_{2,\mathrm{out}}, \hat{a}^{(0)}_{3,\mathrm{out}}, \hat{b}_{1,\mathrm{out}}, \hat{b}_{2,\mathrm{out}})^T$. The matrix M is here given by

$$M = \begin{pmatrix} -\frac{\kappa_1}{2} & 0 & 0 & -ig_{11}e^{i\phi_1} & -ig_{12} \\ 0 & -\frac{\kappa_2}{2} & 0 & -ig_{21}e^{i\phi_2} & -ig_{22} \\ 0 & 0 & -\frac{\kappa_3}{2} & -ig_{31}e^{i\phi_3} & -ig_{32} \\ -ig_{11}e^{-i\phi_1} & -ig_{21}e^{-i\phi_2} & -ig_{31}e^{-i\phi_3} & -\frac{\Gamma_{\mathrm{m},1}}{2} + i\delta_1 & 0 \\ -ig_{12} & -ig_{22} & -ig_{32} & 0 & -\frac{\Gamma_{\mathrm{m},2}}{2} + i\delta_2 \end{pmatrix}, \tag{40}$$

where the cavity dissipation rates are the sum of the external and internal dissipation rates, i.e. $\kappa_i = \kappa_{\mathrm{ex},i} + \kappa_{0,i}$. The matrix L is here

$$L = \begin{pmatrix} \sqrt{\kappa_{\mathrm{ex},1}} & 0 & 0 & \sqrt{\kappa_{0,1}} & 0 & 0 & 0 & 0 \\ 0 & \sqrt{\kappa_{\mathrm{ex},2}} & 0 & 0 & \sqrt{\kappa_{0,2}} & 0 & 0 & 0 \\ 0 & 0 & \sqrt{\kappa_{\mathrm{ex},3}} & 0 & 0 & \sqrt{\kappa_{0,3}} & 0 & 0 \\ 0 & 0 & 0 & 0 & 0 & 0 & \sqrt{\Gamma_{\mathrm{m},1}} & 0 \\ 0 & 0 & 0 & 0 & 0 & 0 & 0 & \sqrt{\Gamma_{\mathrm{m},2}} \end{pmatrix}. \tag{41}$$

Using the input-output relation (supplementary eq. (9)) and the matrix form of the equations of motion (supplementary eq. (6)), we can compute the scattering matrix $S(\omega)$ similarly to supplementary eq. (11).

We choose to operate the circulator in a way that suppresses the propagation in the clockwise direction, i.e. $S_{12}(0) = 0$, $S_{23}(0) = 0$, and $S_{31}(0) = 0$. For this suppression to occur on resonance ($\omega = 0$), δ_1 must scale with $\Gamma_{\mathrm{m},1}$ and δ_2 with $\Gamma_{\mathrm{m},2}$ so we define $\delta_1 = \alpha\Gamma_{\mathrm{m},1}$ and $\delta_2 = \beta\Gamma_{\mathrm{m},2}$. The equations corresponding to $S_{12}(0) = S_{23}(0) = S_{31}(0) = 0$ are

$$-2i\alpha - 2i\beta e^{i(\phi_1-\phi_2)} - \mathcal{C}(1 - e^{i(\phi_1-\phi_3)} - e^{i(\phi_3-\phi_2)} + e^{i(\phi_1-\phi_2)}) - (1 + e^{i(\phi_1-\phi_2)}) = 0, \tag{42}$$

$$-2i\alpha - 2i\beta e^{i(\phi_2-\phi_3)} - \mathcal{C}(1 - e^{i(\phi_2-\phi_1)} - e^{i(\phi_1-\phi_3)} + e^{i(\phi_2-\phi_3)}) - (1 + e^{i(\phi_2-\phi_3)}) = 0, \tag{43}$$

$$-2i\alpha - 2i\beta e^{i(\phi_3-\phi_1)} - \mathcal{C}(1 - e^{i(\phi_3-\phi_2)} - e^{i(\phi_2-\phi_1)} + e^{i(\phi_3-\phi_1)}) - (1 + e^{i(\phi_3-\phi_1)}) = 0. \tag{44}$$

Analysing this set of equations, we see that only two phases are independent. Setting $\phi_1 = 2\pi/3$, $\phi_2 = -2\pi/3$ and $\phi_3 = 0$ leads to a set of fully degenerate equations. We then obtain $S_{13}(0) = S_{32}(0) = S_{21}(0) = 0$ if

$$2\sqrt{3}\beta - 3\mathcal{C} - 1 + i\left(4\alpha - 2\beta + 3\sqrt{3}\mathcal{C} + \sqrt{3}\right) = 0. \tag{45}$$

Solving supplementary eq. (45) with respect to the cooperativity $\mathcal{C}$ gives

$$\mathcal{C} = \frac{2\beta}{\sqrt{3}} - \frac{1}{3} \quad \text{and} \quad \alpha = -\beta. \tag{46}$$

We note that if $\alpha \neq -\beta$, then $\mathcal{C}$ must contain an imaginary part leading to complex coupling strengths, which is inconsistent with their definition as being real. Moreover, $\mathcal{C}$ must be positive (such that $g_{ij} \in \mathbb{R}$) and non-zero (else $g_{ij} = 0$). The lower bound for β is thus given by $1/(2\sqrt{3})$.

We can write the transmission in the counter-clockwise direction ($|S_{13}|^2$, $|S_{32}|^2$ and $|S_{21}|^2$) on resonance as

$$|S_{13}|^2 = \frac{\kappa_{\text{ex},1}\kappa_{\text{ex},3}}{\kappa_1\kappa_3}\frac{1}{(1+\frac{1}{3\mathcal{C}})^2}, \tag{47}$$

$$|S_{32}|^2 = \frac{\kappa_{\text{ex},3}\kappa_{\text{ex},2}}{\kappa_3\kappa_2}\frac{1}{(1+\frac{1}{3\mathcal{C}})^2}, \tag{48}$$

$$|S_{21}|^2 = \frac{\kappa_{\text{ex},2}\kappa_{\text{ex},1}}{\kappa_2\kappa_1}\frac{1}{(1+\frac{1}{3\mathcal{C}})^2}. \tag{49}$$

We find that, in the case of overcoupled cavities $\kappa_i \approx \kappa_{\text{ex},i}$, the transmission approaches unity with increasing cooperativity.

The symmetrised output noise spectra is computed as in the supplementary note 2. In the limit of overcoupled cavities, $\kappa_i \approx \kappa_{\text{ex},i}$, the noise emitted on resonance at each port is given by

$$N = \frac{1}{2} + \frac{3\mathcal{C}}{(3\mathcal{C}+1)^2}(\bar{n}_{\text{m},1} + \bar{n}_{\text{m},2}). \tag{50}$$

In the limit of large cooperativity, the noise contribution from the mechanical oscillators is entirely suppressed, leaving only vacuum noise amounting to half a quantum.

SUPPLEMENTARY REFERENCES

[1] Aspelmeyer, M., Kippenberg, T. J. & Marquardt, F. Cavity optomechanics. *Rev. Mod. Phys.* **86**, 1391–1452 (2014).

[2] Law, C. K. Interaction between a moving mirror and radiation pressure: A hamiltonian formulation. *Phys. Rev. A* **51**, 2537–2541 (1995).

[3] Dobrindt, J. M. & Kippenberg, T. J. Theoretical analysis of mechanical displacement measurement using a multiple cavity mode transducer. *Phys. Rev. Lett.* **104**, 033901 (2010).

[4] Gardiner, C. W. & Collett, M. J. Input and output in damped quantum systems: Quantum stochastic differential equations and the master equation. *Physical Review A* **31**, 3761–3774 (1985).

[5] Clerk, A. A., Devoret, M. H., Girvin, S. M., Marquardt, F. & Schoelkopf, R. J. Introduction to quantum noise, measurement, and amplification. *Rev. Mod. Phys.* **82**, 1155–1208 (2010).
