# Supplementary material for: Nonreciprocal reconfigurable microwave optomechanical circuit
Source: Nat Commun. 2017 Sep 19;8:604. doi: 10.1038/s41467-017-00447-1 (PMC5605717; doi:10.1038/s41467-017-00447-1)
Supplement: Supplementary file 2 — Peer Review File [file 41467_2017_447_MOESM2_ESM.pdf]

Editorial Note: this manuscript has been previously reviewed at another journal that is not operating a transparent peer review scheme. This document only contains reviewer comments and rebuttal letters for versions considered at Nature Communications.

Reviewers' Comments:

Reviewer #1:
Remarks to the Author:
The authors have satisfactorily addressed all the points I have raised in my previous report. The new explanations and the new part involving a 5-mode all-optomechanical circulator have significantly improved the paper. I find the paper an important contribution for the development of on-chip nonreciprocal devices and a clever application of optomechanical systems and therefore I suggest publication of the paper in Nature Communications.

Reviewer #2:
Remarks to the Author:
The revisions performed by the authors has indeed improved the manuscript. I find the current revision of the manuscript suitable for publication in Nature Communications
